# Simulated nitrogen deposition significantly reduces soil respiration in an evergreen broadleaf forest in western China

**DOI:** 10.1371/journal.pone.0204661

**Published:** 2018-09-27

**Authors:** Shixing Zhou, Yuanbin Xiang, Liehua Tie, Bohan Han, Congde Huang

**Affiliations:** College of Forestry, Sichuan Agricultural University, Chengdu, China; Tennessee State University, UNITED STATES

## Abstract

Soil respiration is the second largest terrestrial carbon (C) flux; the responses of soil respiration to nitrogen (N) deposition have far-reaching influences on the global C cycle. N deposition has been documented to significantly affect soil respiration, but the results are conflicting. The response of soil respiration to N deposition gradients remains unclear, especially in ecosystems receiving increasing ambient N depositions. A field experiment was conducted in a natural evergreen broadleaf forest in western China from November 2013 to November 2015 to understand the effects of increasing N deposition on soil respiration. Four levels of N deposition were investigated: control (Ctr, without N added), low N (L, 50 kg N ha^−1^·a^−1^), medium N (M, 150 kg N ha^−1^·a^−1^), and high N (H, 300 kg N ha^−1^·a^−1^). The results show that (1) the mean soil respiration rates in the L, M, and H treatments were 9.13%, 15.8% (*P* < 0.05) and 22.57% (*P* < 0.05) lower than that in the Ctr treatment (1.56 ± 0.13 μmol·m^−2^·s^−1^), respectively; (2) soil respiration rates showed significant positive exponential and linear relationships with soil temperature and moisture (*P* < 0.01), respectively. Soil temperature is more important than soil moisture in controlling the soil respiration rate; (3) the Ctr, L, M, and H treatments yielded *Q*_10_ values of 2.98, 2.78, 2.65, and 2.63, respectively. N deposition decreased the temperature sensitivity of soil respiration; (4) simulated N deposition also significantly decreased the microbial biomass C and N, fine root biomass, pH and extractable dissolved organic C (*P* < 0.05). Overall, the results suggest that soil respiration declines in response to N deposition. The decrease in soil respiration caused by simulated N deposition may occur through decreasing the microbial biomass C and N, fine root biomass, pH and extractable dissolved organic C. Ongoing N deposition may have significant impacts on C cycles and increase C sequestration with the increase in global temperature in evergreen broadleaf forests.

## Introduction

The amount of carbon dioxide (CO_2_) released through soil respiration can reach 68–100 Pg C each year [1], and slight changes in soil respiration can significantly alter CO_2_ concentrations in the atmosphere [2, 3]. It is well known that a complex array of biotic and environmental factors, such as temperature, precipitation, soil moisture, root biomass, oxygen, changes in microbial C use efficiency and substrate availability, affect soil respiration [4–7]. Global changes (such as global warming, elevated CO_2_ and nitrogen (N) deposition) may also substantially mediate soil respiration [7–9].

Human activities such as fossil fuel combustion and synthetic fertilizer application have dramatically accelerated the fixation and global movement of reactive N since the Industrial Revolution [10, 11]. At present, the amount of active N produced by human activities exceeds the amount of active N produced by natural land processes [10]. After Europe and the United States, China is considered the third-largest N deposition region in the world [10, 12, 13]. Excess N deposition has caused a series of ecological problems, such as plant growth alterations, soil acidification, soil nutrient storage imbalances, and changes to other ecosystem functions [10, 11, 14]. Many studies in terrestrial ecosystems have reported that N deposition generally promotes plant growth; however, N deposition may have negative effects in some N-rich ecosystems [11, 15–17]. The C balance in terrestrial ecosystems is determined by C fixation (e.g., plant growth) and C emission (e.g., soil respiration) processes. Therefore, the direction and extent of the C balance can be significantly affected by N deposition via soil respiration alterations [18].

**Fig 1:**
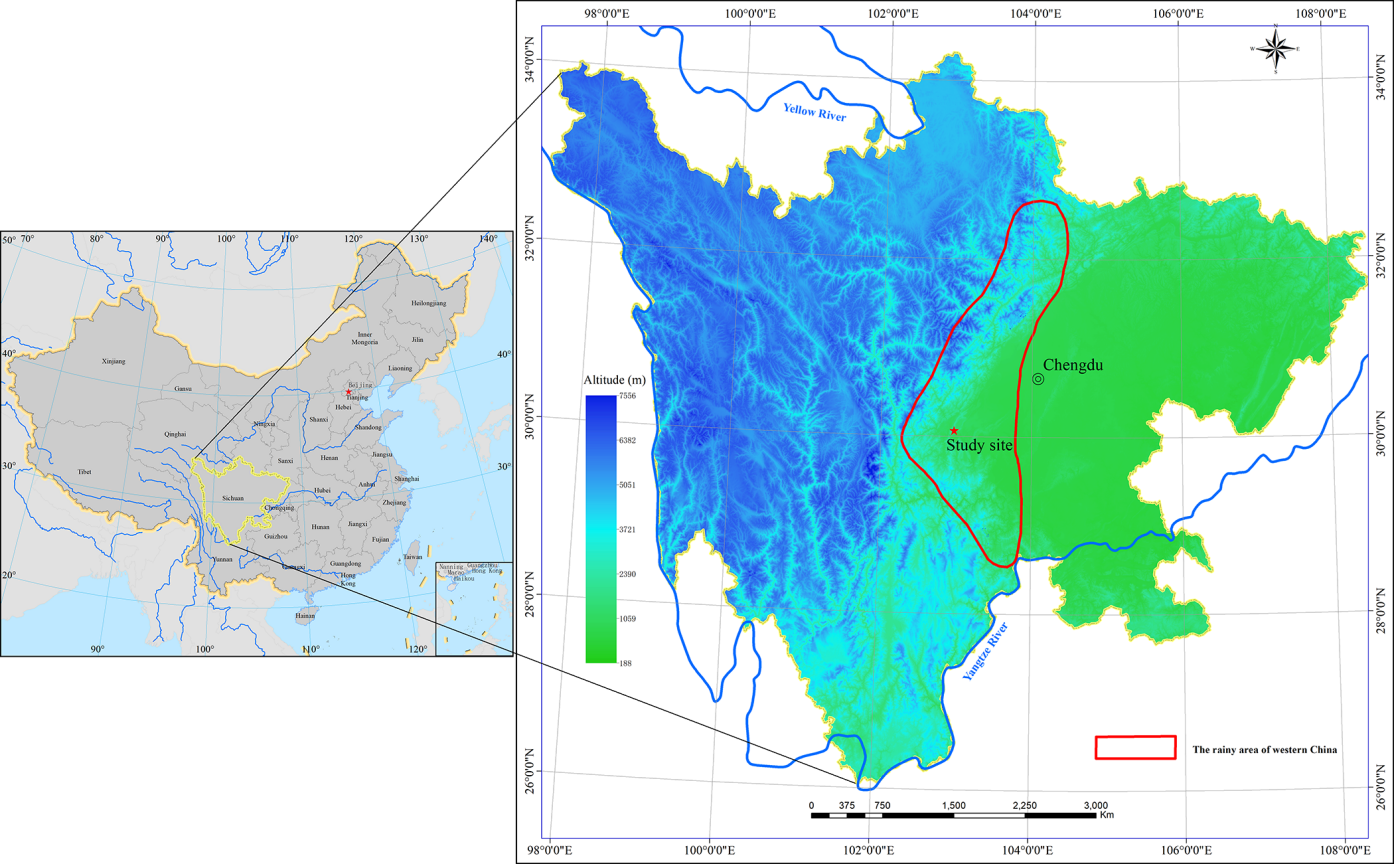
Locations of the rainy area of western China and the study site. The map was generated with ArcGIS version 10.0 (ESRI, Redlands, CA, USA, http://desktop.arcgis.com/en/arcmap/).

The rainy area of western China is a large ecotone on the western edge of the Sichuan Basin that spans 400–450 km north to south and 50–70 km east to west with an area of approximately 25,000 km^2^ (Fig 1) [19–21]. Due to the high elevation and the effect of the East Asian Monsoon and Indian Monsoon, warm moist air from the Sichuan Basin is readily condensed into rain on the western edge of the basin, resulting in abundant rainfall [19, 22]. Due to the climate and topography, this area receives considerable N deposition from the Sichuan Basin [13, 23]. The average atmospheric wet N deposition in this area was approximately 95 kg N·ha^-1^ from 2008 to 2010 [13, 24, 25], which is much higher than that in other areas of China [13]. Numerous manipulative experiments have demonstrated that the response of soil respiration to N deposition can be complex. Previous studies have indicated that N deposition suppressed [26–28], promoted [29–31], or had no effect [32–34] on soil respiration. Although soil respiration has been widely explored, most previous studies have been conducted in temperate forest ecosystems (with relatively low rates of ambient N deposition) [18, 28]. The effects of N deposition on soil respiration in ecosystems that are impacted by increasing ambient N deposition (especially tropical and subtropical forest ecosystems) are not well understood. To understand the responses of soil respiration to increasing N deposition, especially under high levels of ambient N deposition, we performed a manipulative N deposition field experiment in a natural evergreen broadleaf forest (subtropical forest) in the rainy area of western China. It has been hypothesized that an increase in N concentration possibly increases soil respiration and microbial activity in N-limited forest ecosystems [18, 27, 30, 35]. Moreover, we previously reported that the natural evergreen broadleaf forests in the rainy area of western China are N-rich forest ecosystems, and N deposition was found to significantly decrease litter decomposition [20, 36]. Considering these findings, we hypothesized that simulated N deposition likely reduces soil respiration through the combined negative effects on plant and microbial activities in these N-rich subtropical forests, especially under conditions of high atmospheric N deposition.

## Materials and methods

### Ethics statement

All researches were in compliance with laws in People’s Republic of China, no specific permissions were required for these locations or activities, and measurements on humans, animals and endangered or protected plants were not involved in our work.

### Site description

The study was conducted in a natural evergreen broadleaf forest at the Bi Feng Gorge Scenic Spot, Ya’an City, Sichuan Province (30°03’N, 102°59’E, 1,170 m above sea level, Fig 1). The region is located in a subtropical moist forest zone with a monsoon climate [18, 20, 21]. The mean annual precipitation is approximately 1700 mm (average from 1961 to 2011), with approximately 60% falling from July to September [37], and the mean annual temperature is 16.2°C (25.4°C in July and 6.1°C in January) [20, 21, 36]. The soil is classified as a Lithic Dystrudepts (according to USDA Soil Taxonomy), derived from purple sandstone and shale, with a depth of more than 60 cm. The mean concentrations of total C, total N, nitrate N and ammonium N in the soil (0–20 cm depth) are 17.17 ± 1.54 g·kg^-1^, 1.14 ± 0.13 g·kg^-1^, 9.45 ± 1.03 mg·kg^-1^ and 6.87 ± 0.56 mg·kg^-1^, respectively, and the pH is 6.21 ± 0.13 (November 2013) [20, 21, 36]. The dominant tree species are *Schima superba*, *Machilus pingii*, *Rhus succedanea*, *Lithocarpus hancei*, *Acer davidii*, *Pittosporum tobira*, *Machilus lichuanensis*, *Eurya japonica*, *Symplocos botryantha*, *Cinnamomum cassia*, *Lithocarpus megalophyllus*, *Acer sinense*, and *Camellia japonica* [20, 21, 36].

### Experimental design

Twelve plots (3 × 3 m) at 3 m intervals were randomly established in a representative natural evergreen broadleaf forest at the Bi Feng Gorge Scenic Spot in October 2013 [20, 21, 36]. Four levels of N addition with three replicates were established: control (Ctr, without N added), low N deposition (L, 50 kg N ha^−1^·a^−1^), medium N deposition (M, 150 kg N ha^−2^·a^−1^), and high N deposition (H, 300 kg N ha^−1^·a^−1^) [20, 21, 36]. From November 2013 to November 2015, ammonium nitrate (NH_4_NO_3_) was added as a fertilizer twice each month. For each application, the fertilizer was weighed, dissolved in 2 L of water, and evenly sprayed back and forth at a height of 50 cm above the ground with a sprayer. The control plot received 2 L of distilled water without [20, 36].

### Soil respiration rate measurements

In October 2013, three polyvinyl chloride collars (20 cm inside diameter and 12 cm height) were randomly installed into the soil to a depth of 7 cm in each plot. From December 2013 to November 2015, the soil respiration rate (μmol·m^−2^·s^−1^) was measured monthly using an LI-8100 automated soil CO_2_ flux system (LI-COR Inc., USA). All living plants inside the collars were clipped at least 24 h before soil respiration measurements were collected to eliminate the effect of aboveground plant respiration. All soil respiration measurements were performed between 9:00 and 18:00 (local time) on each measurement date at 3 h intervals, and the measurements were performed four times per measurement day [18, 26]. The average of the four measurements was calculated as the soil respiration rate for each month.

### Soil temperature and moisture measurements

Soil temperature (°C) at a soil depth of 10 cm was measured by inserting a soil temperature probe connected to the LI-8100 near the soil CO_2_ flux chamber while soil respiration was being monitored. Simultaneously, a time-domain reflectometer (mini Trase 6050X3K1, ICT, USA) with probes (0–15 cm depth) was used to measure the volumetric soil moisture (%).

### Fine root biomass and soil biochemical characteristic measurements

In November 2015, soil samples (0–20 cm) were collected using a stainless steel soil corer (6 cm in diameter and 20 cm in depth). Three cores were randomly collected from each plot, thoroughly mixed and then divided into two subsamples, one for fine root biomass (subsample 1) and one for the biochemical characteristic (subsample 2) measurements. Fine roots were separated from soils (subsample 1) by washing and sieving with a 0.25 mm sieve. Then, the roots were washed on a sieve, separated, dried at 60°C for 48 h and weighed [38]. The samples in subsample 2 were passed through a 2 mm mesh sieve and used to determine the soil extractable dissolved organic C (DOC) and microbial biomass C and N (MBC and MBN). The soil samples were extracted with distilled water (soil:water ratio, 2:1), shaken for 0.5 h (170 rpm) at 25°C, and centrifuged for 20 min at 3500 rpm; the supernatant was filtered through a membrane (0.45 μm, Millipore, Xingya Corporation, Shanghai, China) into a plastic bottle [39]. Then, the DOC concentrations were determined using a total C and N analyzer (Shimadzu model TOC-V_CPH_ +TNM-1, Kyoto, Japan). In addition, MBC and MBN were measured using the chloroform fumigation extraction technique via the total C and N analyzer. The soil pH value was determined by a glass electrode in aqueous extracts (pH-H_2_O).

### Data analysis

The relationships between soil respiration rate, soil temperature and soil moisture were determined using linear and nonlinear regression models [28, 40, 41]. We fitted the soil respiration rate and soil temperature to an exponential function model: *R*_*S*_ = α × e^β*t*^. *Q*_10_ was obtained from the following coefficient: *Q*_10_ = e^10β^. In the preceding equations, *R*_*S*_ is the soil respiration rate (μmol·m^−2^·s^−1^), *t* denotes the soil temperature (°C), and β indicates the temperature response coefficient [29, 42]. The soil respiration rate and soil moisture were fitted to the following linear equation: *R*_*S*_ = a*W* + b, where *W* is the soil moisture (%). We use the model: *R*_*S*_ = a × e^b*t*^ + c*W* to determine the relationship between soil respiration rate, soil temperature and soil moisture [40].

A repeated-measures analysis of variance (RM-ANOVA) with N deposition as the between-subject effect and measuring time as the within-subject effect was used to analyze the changes in soil respiration, soil temperature and soil moisture. The data were adjusted using the Greenhouse-Geisser method when Mauchly’s test of sphericity was not fulfilled. A one-way analysis of variance (ANOVA) with a least significant difference (LSD) test was used to inspect the differences in soil MBC, MBN, DOC, pH and fine root biomass among all treatments at the end of the experiment. The data were inspected for homogeneity of variance using a Levene test before ANOVA and log_10_-transformed if required. All statistical analyses were performed using SPSS 20.0 (SPSS Inc., USA) for Microsoft Windows.

## Results

### Soil temperature and moisture

**Fig 2:**
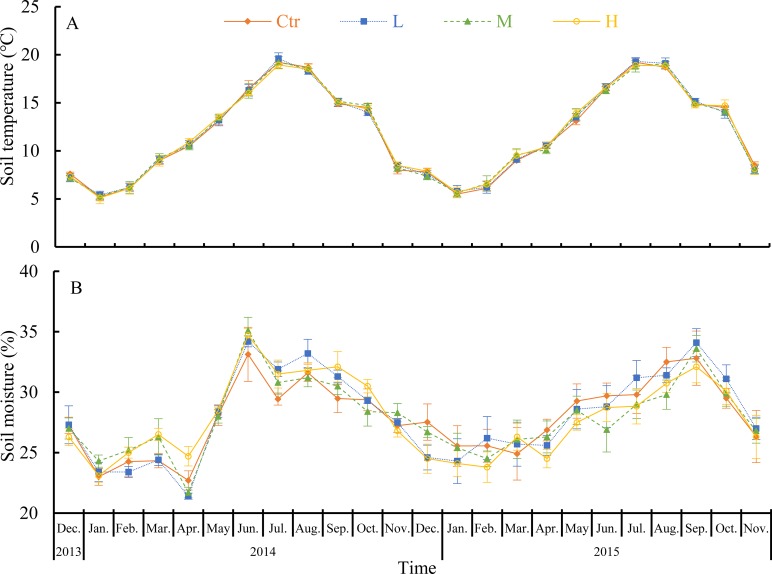
Monthly variations in soil temperature and soil moisture from December 2013 to November 2015. Error bars represent standard deviations of the means (*n* = 3). Ctr (control, without N added), L (low N deposition, 50 kg N ha^−1^·a^−1^), M (medium N deposition, 150 kg N ha^−2^·a^−1^), and H (high N deposition, 300 kg N ha^−1^·a^−1^).

Soil temperature at a soil depth of 10 cm followed a strong seasonal pattern (Fig 2A), whereas soil moisture varied little with season (Fig 2B). Soil temperature in the control ranged from 5.2 ± 0.4°C (January 2013) to 19.2 ± 0.4°C (July 2015, Fig 2A); soil moisture ranged from 22.7 ± 0.8% (April 2014) to 33.1 ± 2.2% (June 2014, Fig 2B). RM-ANOVA showed that soil temperature and moisture did not significantly differ among the treatments (*P* = 0.32 and 0.99, respectively; Fig 2 and Table 1).

**Table 1:** Results of the repeated-measures ANOVAs of soil respiration rate (log transformed), soil temperature and soil moisture (Mean ± SD).

Treatments	Soil respiration rate (μmol·m^−2^·s^−1^)	Soil temperature (°C)	Soil moisture (%)
Ctr	1.55 ± 0.05 a	12.0 ± 0.2 a	27.9 ± 0.7 a
L	1.41 ± 0.12 ab	12.1 ± 0.5 a	28.1 ± 0.9 a
M	1.31 ± 0.09 b	12.1 ± 0.3 a	27.9 ± 1.0 a
H	1.20 ± 0.07 b	12.0 ± 0.4 a	27.9 ± 0.8 a
*N effect*	*P* < 0.01	*P* = 0.32	*P* = 0.99
*Time effect*	*P* < 0.01	*P* < 0.01	*P* < 0.01
*N × Time effect*	*P* < 0.01	*P* = 0.99	*P* < 0.01

Different letters indicate significant differences among treatments with a > b > c (RM-ANOVA with Fisher’s LSD test, α = 0.05). Ctr (control, without N added), L (low N deposition, 50 kg N ha^−1^·a^−1^), M (medium N deposition, 150 kg N ha^−2^·a^−1^), and H (high N deposition, 300 kg N ha^−1^·a^−1^).

### Soil respiration

**Fig 3:**
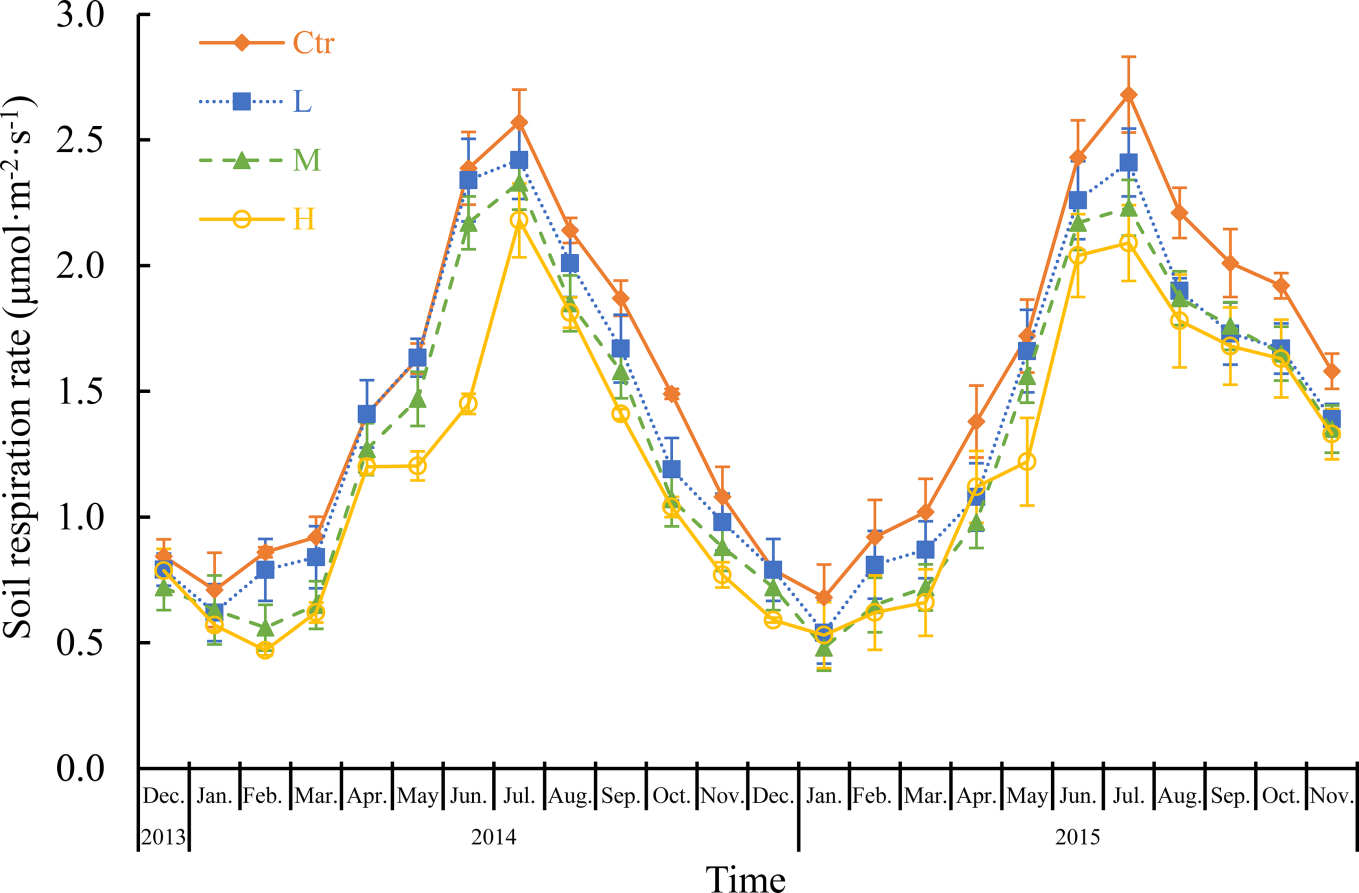
Monthly dynamics of soil respiration rate from December 2013 to November 2015. Error bars represent standard deviations of the means (*n* = 3). Treatment abbreviations are provided in Fig 1.

Soil respiration rate followed a clear seasonal pattern during the two year study period, and it was higher in summer and lower in winter (Fig 3). In the control treatment, the maximum soil respiration occurred in July 2014 (2.68 ± 0.15 μmol·m^−2^·s^−1^), and the minimum occurred in January 2014 (0.64 ± 0.13 μmol·m^−2^·s^−1^) (Fig 3). The mean soil respiration rate in the control treatment was 1.56 ± 0.13 μmol·m^−2^·s^−1^ (Table 2).

**Table 2:** Parameters of different regression models between soil respiration rate, soil temperature and soil moisture.

*R*_*S*_ = α × e^β*t*^								
Treatment	*α*	*β*		*R*^*2*^		*P*		*Q*_*10*_
Ctr	0.34	0.1092		0.77		< 0.01		2.98
L	0.37	0.1024		0.72		< 0.01		2.78
M	0.39	0.0973		0.73		< 0.01		2.65
H	0.33	0.0968		0.82		< 0.01		2.63
*Rs* = *aW* + *b*								
Treatment	*a*		*b*		*R*^*2*^		*P*	
Ctr	14.80		−2.58		0.56		< 0.01	
L	12.79		−2.18		0.61		< 0.01	
M	13.33		−2.42		0.47		< 0.01	
H	12.87		−2.11		0.51		< 0.01	
*R*_*S*_ *= c × e*^*dt*^ *+ fW*								
Treatment	*c*	*d*		*f*		*R*^*2*^		*P*
Ctr	0.19	0.13		1.60		0.80		< 0.01
L	0.10	0.15		2.48		0.79		< 0.01
M	0.13	0.14		1.89		0.80		< 0.01
H	0.72	0.07		-1.85		0.83		< 0.01

*Rs*: soil respiration rate (μmol·m^−2^·s^−1^), *t*: soil temperature (°C), *W*: soil moisture (%). Treatment abbreviations are provided in Table 1.

Throughout the study period, the mean soil respiration rates in the L, M, and H treatments were 1.41 ± 0.12, 1.31 ± 0.09 and 1.20 ± 0.07 μmol·m^−2^·s^−1^, respectively (Table 1), which were 9.13%, 15.8% (*P* < 0.05) and 22.57% (*P* < 0.05) lower than those in the Ctr treatment (1.56 ± 0.13 μmol·m^−2^·s^−1^), respectively. Simulated N deposition significantly reduced soil respiration rates in the M and H treatments compared with those in the Ctr and L treatments (*P* < 0.05, Fig 3 and Table 1).

### Relationships among soil respiration, soil temperature and moisture

**Fig 4:**
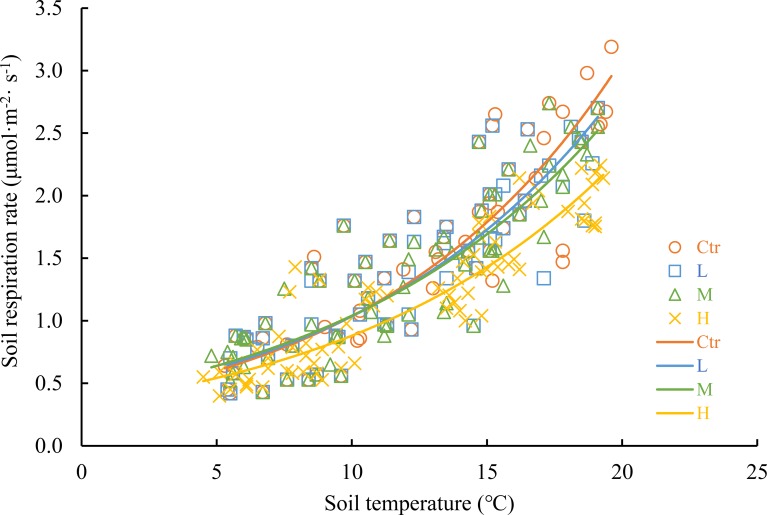
Relationships between soil respiration rate and soil temperature. Treatment abbreviations are provided in Fig 2.

**Fig 5:**
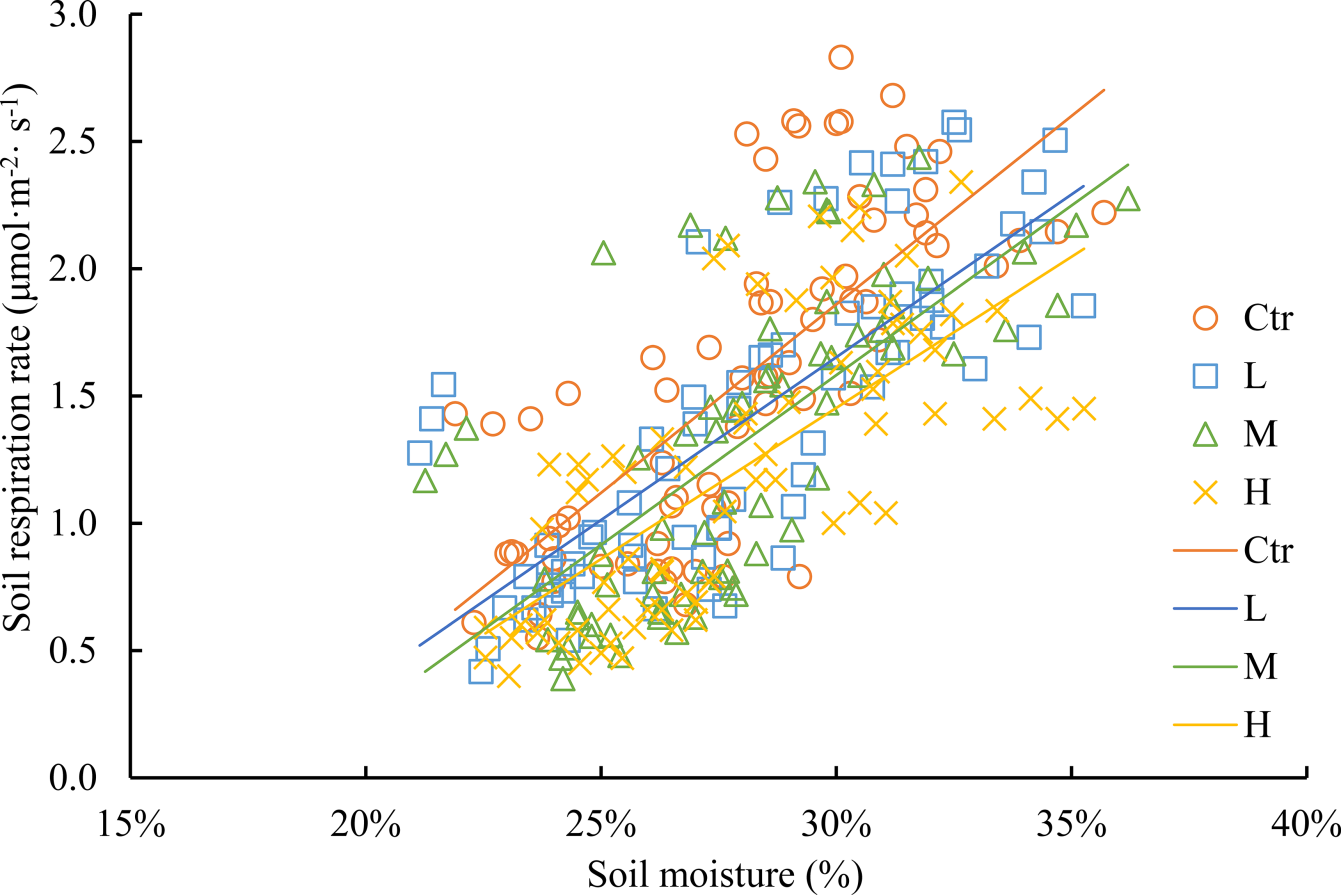
Relationships between soil respiration rate and soil moisture. Treatment abbreviations are provided in Fig 2.

Relationship between soil respiration rate and soil temperature was fitted with an exponential model, and soil temperature explained 72–82% of the monthly variations in soil respiration (*P* < 0.01, Fig 4 and Table 2). There was a highly significant linear relationship between soil respiration and soil moisture; soil moisture explained 47–61% of the monthly variations in soil respiration (*P* < 0.01, Fig 5 and Table 2). The model that combined an exponential component (soil respiration rate and soil temperature) with a linear component (soil respiration rate and soil moisture) yielded higher *R*^2^ values than the exponential or linear model alone (Table 2); adding soil moisture to models with only soil temperature increased the *R*^2^ values from 0.72–0.82 to 0.79–0.83. Soil temperature was more important than soil moisture in controlling the soil respiration rate in this natural evergreen broadleaf forest.

The *Q*_10_ values of the L, M, and H treatments were 0.2, 0.13, and 0.15 lower than that in the Ctr treatment (2.98), respectively (Table 2). Simulated N deposition decreased the temperature sensitivity of soil respiration in this natural evergreen broadleaf forest.

### MBC, MBN, DOC, pH and fine root biomass

**Fig 6:**
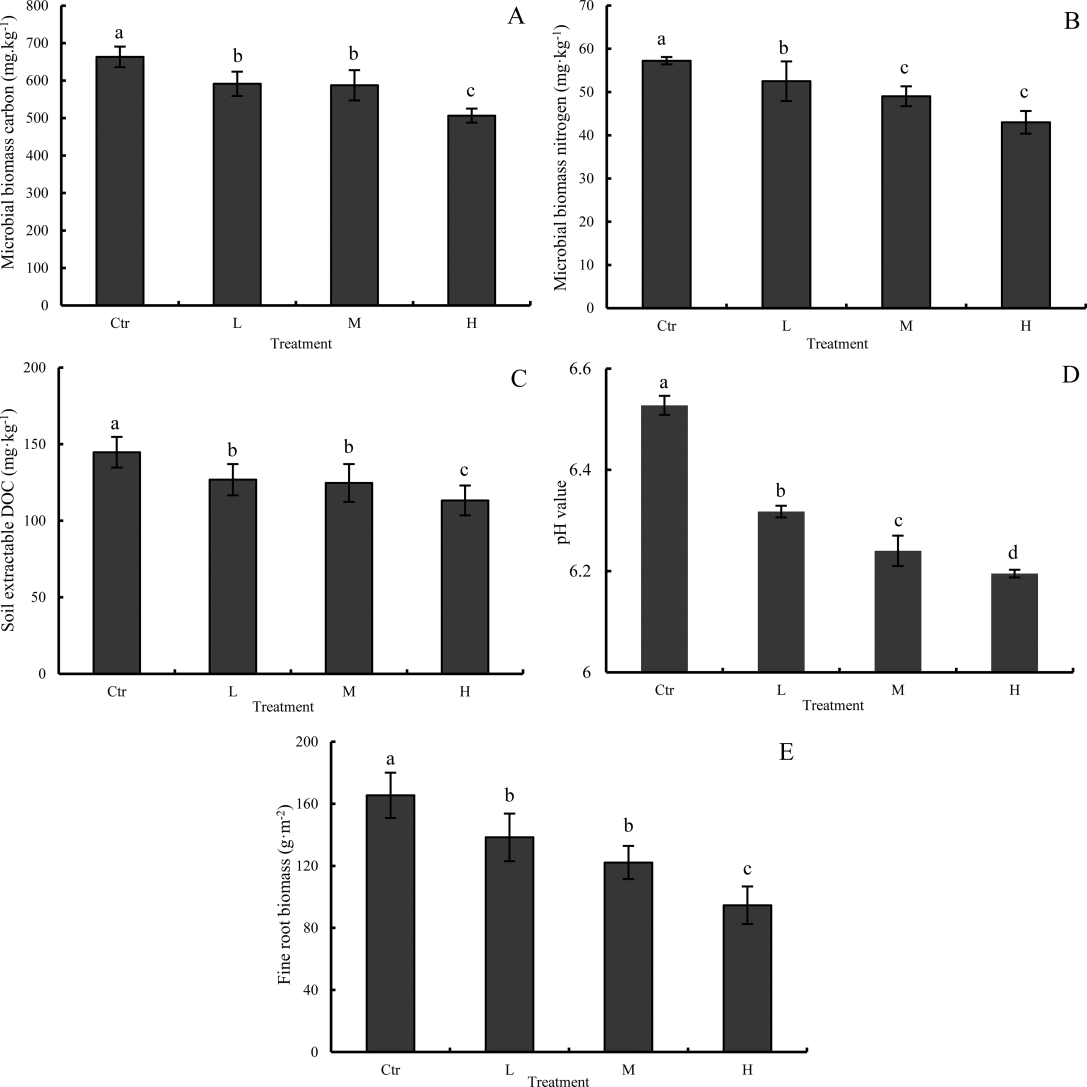
**MBC (A), MBN (B), DOC (C), pH (D) and fine root biomass (E) under different treatments in November 2015.** Different letters denote significant differences (one-way ANOVA with Fisher’s LSD test, *P* < 0.05) between treatments. Error bars represent standard deviations of the means (*n* = 3). Treatment abbreviations are provided in Fig 2.

Simulated N deposition significantly affected MBC, MBN, DOC, pH and fine root biomass (S1 Table and Fig 6). After 2 years of simulated N deposition, the soil MBC values in the L, M, and H treatments were 10.84% (*P <* 0.05), 11.42% (*P <* 0.05), and 23.61% (*P <* 0.05) lower than that in the Ctr treatment, respectively (663.30 ± 27.58 mg·kg^-1^, Fig 6A). Moreover, the MBN values in the L, M and H treatments were 8.25% (*P <* 0.05), 14.33% (*P <* 0.05), and 24.89% (*P <* 0.05) lower than that in the Ctr treatment, respectively (57.23 ± 3.58 mg·kg^-1^, Fig 6B). The extractable DOC values in the L, M, and H treatments were 10.84% (*P <* 0.05), 11.42% (*P <* 0.05), and 23.61% (*P <* 0.05) lower than that in the Ctr treatment, respectively (144.72 ± 10.01 mg·kg^-1^, Fig 6C). The pH values in the L, M, and H treatments were 6.53% (*P <* 0.05), 4.44% (*P <* 0.05), and 5.13% (*P <* 0.05) lower than that in the Ctr treatment, respectively (6.53 ± 0.02, Fig 6D). The fine root biomass values in the L, M, and H treatments were 16.39% (*P <* 0.05), 26.17% (*P <* 0.05), and 42.83% (*P <* 0.05) lower than that in the Ctr treatment, respectively (165.43 ± 14.63 g·m^-2^, Fig 6E). Thus, all simulated N deposition treatments significantly decreased the pH, fine root biomass and concentrations of MBC, MBN, and DOC. (*P <* 0.05).

## Discussion

In our study, we found that soil respiration followed a clear seasonal pattern in this natural evergreen broadleaf subtropical forest, and it was higher in summer and lower in winter (Fig 3). The clear seasonal pattern of soil respiration can be reflected by similar seasonal changes in soil temperature (Fig 2A). Many previous studies found that soil temperature and soil moisture were two of the most important environmental parameters that controlled the temporal variations in soil respiration [3, 4, 26, 28, 40]. In our study, we found that soil temperature explained 72–82% of the monthly variations, while soil moisture explained 47–61% of the monthly variations in soil respiration; adding soil moisture to a model with only soil temperature increased the *R*^2^ values from 0.72–0.82 to 0.79–0.83 (Table 2). Soil temperature was found to be more important than soil moisture in controlling soil respiration in our study. Generally, in most forest ecosystems, soil temperature is the dominant factor in soil respiration [26, 43]. In our study, this finding may be due to the relatively high annual rainfall in this zone (1700 mm) and the strong water-holding capacity of the soil during the monitoring period (soil moisture ranged from 22.7 ± 0.8% to 33.1 ± 2.2%, Fig 2B). As a result, relatively high soil moisture was maintained, and the humidity conditions in the soil were suitable for plant root and microbial activities. However, soil temperature can directly affect plants and microbial activities and indirectly change soil moisture and the amount and quality of soil organic matter [44, 45]. Therefore, the soil temperature becomes the dominant factor that influences soil respiration in this natural evergreen broadleaf forest.

*Q*_10_ values reflect the sensitivity of the soil respiration rate to temperature and are commonly used to estimate soil C emissions [46–48]. The *Q*_10_ value in the control treatment in our study was 2.98 (Table 2). This value is similar to that measured in a tundra forest (3.05) [49], but it is lower than that in a temperate hardwood forest (3.9) [50] and higher than those in a bamboo ecosystem (2.48) [23] and a tropical moist forest (2.6) [28]. Furthermore, we also found simulated N deposition had a negative effect on the *Q*_10_ values, and the values of the L, M, and H treatments were 0.2, 0.13, and 0.15 lower than that of the Ctr treatment (2.98), respectively (Table 2). This result suggests that N deposition changes the temperature control on soil respiration; however, the effects were slight. This result is consistent with the results of studies by Mo et al. [28] in a tropical old-growth monsoon evergreen broadleaf forest, Tu et al. [18] in a subtropical bamboo ecosystem and Sun et al. [26] in a temperate larch forest. A reduction in the *Q*_*10*_ values may result from an increase in the stability of soil organic C via the formation of stabilizing organic matter [27] or the decrease in allocation of photosynthates to the rhizosphere [26, 51] under N-rich conditions.

As intended, simulated N deposition significantly decreased soil respiration, and this was most pronounced in the M and H treatments (Fig 3 and Table 1), which induced decreases in CO_2_ transfer from the soil to the atmosphere. Recently, a study conducted in a secondary evergreen broad-leaved forest in the rainy area of western China also found that N deposition (150 kg N ha^−1^·a^−1^) significantly decreased soil respiration [22, 52]. These findings agree with several studies that revealed that N deposition leads to short-term decreases in soil respiration [26–28, 53]; however, these results are contradictory to the results of other studies [18, 23, 54–56]. Generally, soil respiration comprises both heterotrophic respiration from fauna and free-living soil microorganisms (actinomycetes, bacteria, fungi and protozoans) and rhizosphere respiration from mycorrhizae, roots and other microorganisms associated with root systems [26, 52, 57, 58]. Soil respiration can also be roughly divided into microbial respiration and root respiration on the basis of CO_2_ emissions [23, 58]. Therefore, the effects of N deposition on soil respiration can be classified into two aspects: root respiration and microbial respiration. The decrease in soil respiration due to the simulated N deposition in our study may be related to the following two mechanisms.

First, N deposition may decrease rhizosphere respiration from plant roots. In general, plant root respiration contributes a large proportion to soil respiration. Previous studies found that fine root biomass is significantly and positively correlated with soil respiration [52, 59, 60]. Similar to the results of this study, many previous studies found that N deposition can decrease root biomass, which leads to less input from belowground roots to the soil [22, 27, 28, 61]. In our study, we found that fine root biomass was significantly reduced by simulated N deposition (*P* < 0.05, Fig 6E). An increase in soil N availability may induce plants to allocate a greater proportion of biomass to aboveground organs and decrease the allocation of photosynthates to the rhizosphere [26, 51], resulting in a reduction in underground biomass [62] and thus leading to decreased root respiration in the soil.

Second, N deposition may reduce heterotrophic respiration from the microbial community. Soil microbial respiration depends on soil temperature, moisture, microbial biomass and extracellular enzyme activities, as well as substrate quality and quantity [18, 27, 28, 52]. In our study, we found that N deposition significantly decreased the pH (Fig 6D). Given that soil pH is crucial to enzyme functioning [27, 63], soil acidification caused by N deposition could have a detrimental effect on microbial activity, thus leading to reduced heterotrophic respiration from the microbial community. Moreover, in our previous papers, we have reported that simulated N deposition significantly decreased the number of culturable bacteria and fungi [64] at our study sites. In this study, after two years of simulated N deposition, the MBC and MBN values in the L, M, and H treatments were significantly lower than those in the Ctr treatment (Fig 6A and 6B). Simulated N deposition significantly decreased MBC and MBN in our study, and this result is in line with previous studies in other forest ecosystems [9, 22, 26, 27, 65]. Furthermore, we also found that N deposition significantly decreased the concentration of DOC (Fig 6C). The reduction in MBC, MBN and DOC caused by N deposition indicates that simulated N deposition might have strongly decreased heterotrophic respiration in our study.

## Conclusions

Our results highlight that simulated N deposition significantly affects soil respiration, and this was most pronounced in the M and H treatments despite the high rates of ambient N deposition. The negative impacts of N deposition on soil respiration may occur via negative impacts to plant and microbial activities (i.e., decreasing the MBC, MBN, fine root biomass, and DOC contents). Furthermore, N deposition also decreased the *Q*_10_ value. Ongoing N deposition may have a potentially significant impact on C cycles and could increase C sequestration with the increase in global temperature, thereby threatening the long-term persistence of evergreen broadleaf forest ecosystems in western China.

## Supporting information

S1 Table ANOVA table for the effects of N deposition on MBC, MBN, DOC, pH and fine root biomass. *SS* = sum of squares; *d*.*f*. = degrees of freedom; *MS =* mean square; *F* = F statistics; *P* = statistics significance.
(PDF)
