# Supplementary material for: Simulated nitrogen deposition significantly reduces soil respiration in an evergreen broadleaf forest in western China
Source: PLoS One. 2018 Sep 27;13(9):e0204661. doi: 10.1371/journal.pone.0204661 (PMC6160095; doi:10.1371/journal.pone.0204661)
Supplement: S1 Table — SS = sum of squares; d.f. = degrees of freedom; MS = mean square; F = F statistics; P = statistics significance. (PDF) [file pone.0204661.s001.pdf]

S1 Table. ANOVA table for the effects of N deposition on MBC, MBN, DOC, pH and fine root biomass. *SS* = sum of squares; *d.f.* = degrees of freedom; *MS* = mean square; *F* = F statistics; *P* = statistics significance.

	SS	*d.f.*	*MS*	*F*	*P*
MBC	36864.31	3	12288.10	13.00	< 0.01
MBN	323.74	3	107.92	12.800	< 0.01
DOC	1527.98	3	509.33	4.51	< 0.05
pH	0.20	3	0.07	180.33	< 0.01
fine root biomass	7922.42	3	2640.81	14.93	< 0.01
